# A LIFE MORE ORDINARY: CREATING DEMENTIA-FRIENDLY SPACES THROUGH THE PERFORMATIVE ARTS

**DOI:** 10.1093/geroni/igz038.1965

**Published:** 2019-11-08

**Authors:** Christine Milligan

**Affiliations:** Lancaster University, Lancaster, United Kingdom

## Abstract

The concept of dementia friendly communities emerged from the ‘age-friendly’ movement that has been supported by the WHO for some time. This recognizes that like most older people, those with dementia desire to remain in their own homes, and their own communities, for as long as possible. But it also recognizes the significant socio-environmental challenges this can present. Transforming attitudes to dementia, supporting family and friend caregivers, and promoting meaningful participation for all in the community are essential to the success of any such movement. This paper draws on a qualitative evaluation of one such programme that has sought to develop innovative dementia friendly spaces through the arts and arts performance. Focusing on the Dukes Theatre in Lancaster, UK and its partner theatres and cinemas, I discuss how these venues have, over a three year period, developed spaces in which both people with dementia and their family carers can continue to meaningfully participate in ordinary everyday activities that can be crucial to maintaining the sense of belonging and partnership that is often lost as the dementia journey progresses. Whilst efforts to implement change at the city and community level are laudable, I suggest that it is perhaps at the micro-scale of individual places that we are most likely to successfully stimulate change.

